# Bioprocess monitoring applications of an innovative ATR-FTIR spectroscopy platform

**DOI:** 10.3389/fbioe.2024.1349473

**Published:** 2024-05-28

**Authors:** Loren Christie, Samantha Rutherford, David S. Palmer, Matthew J. Baker, Holly J. Butler

**Affiliations:** ^1^ Dxcover Ltd., Glasgow, United Kingdom; ^2^ Pure and Applied Chemistry, University of Strathclyde, Glasgow, United Kingdom; ^3^ School of Medicine and Dentistry, University of Central Lancashire, Preston, United Kingdom

**Keywords:** fourier transform infrared spectroscopy (FTIR), bioprocess monitoring, process analytical technology (PAT), metabolite quantification, bioprocessing

## Abstract

Pharmaceutical manufacturing is reliant upon bioprocessing approaches to generate the range of therapeutic products that are available today. The high cost of production, susceptibility to process failure, and requirement to achieve consistent, high-quality product means that process monitoring is paramount during manufacturing. Process analytic technologies (PAT) are key to ensuring high quality product is produced at all stages of development. Spectroscopy-based technologies are well suited as PAT approaches as they are non-destructive and require minimum sample preparation. This study explored the use of a novel attenuated total reflection Fourier transform infrared (ATR-FTIR) spectroscopy platform, which utilises disposable internal reflection elements (IREs), as a method of upstream bioprocess monitoring. The platform was used to characterise organism health and to quantify cellular metabolites in growth media using quantification models to predict glucose and lactic acid levels both singularly and combined. Separation of the healthy and nutrient deficient cells within PC space was clearly apparent, indicating this technique could be used to characterise these classes. For the metabolite quantification, the binary models yielded *R* ^2^ values of 0.969 for glucose, 0.976 for lactic acid. When quantifying the metabolites in tandem using a multi-output partial least squares model, the corresponding *R* ^2^ value was 0.980. This initial study highlights the suitability of the platform for bioprocess monitoring and paves the way for future in-line developments.

## 1 Introduction

Bioprocessing describes the way in which biological products can be manufactured from living organisms and is a fundamental component of the pharmaceutical industry. Organisms can be genetically modified to express active pharmaceutical ingredients, including products such as insulin, monoclonal antibodies and vaccines, that can be used in the pharmaceutical industry (Siew and Zhang, 2021; Kelley, 2009). Upstream bioprocessing describes the multi-step process where an organism is grown at large scale volumes, requiring significant resource and expense to complete. A major risk is that the product yield is insufficient, often due to cell health or contamination. Large scale production of biologics is a high-cost process that requires precise monitoring (Satzer et al., 2022).

Monitoring organism growth during bioproduction is critical to the success of the culture. Process analytical technologies (PAT) focus on the measurement of key performance indicators, or critical process parameters, to assess cell growth (Rathore et al., 2010). These measured factors include pH, temperature, dissolved oxygen, and cellular metabolites. Bioreactors are carefully monitored during cell expansion to measure key performance indicators, either by on-line (analysing within the production environment), at-line (collection of samples from production environment and analysed nearby the production environment), in-line measurements (similar to on-line measurements but relates to the use of sensors or monitors positions within the production line), or finally off-line (where samples are removed and analysed separately from the production environment, in a controlled laboratory) (Zhao et al., 2015).

During cell culture expansion, glucose levels are depleted in the cell medium as it serves as the main carbon source to the organisms. Conversely, lactic acid levels rise as by-products of anaerobic respiration (Tsao et al., 2005). An insufficient supply of glucose may inhibit cell growth and reduce yield of a bioreactor, and an accumulation of lactic acid can lead to adverse decrease in pH which can be toxic to cell lines. In large scale and continuous cultures these key parameters must be closely monitored to ensure optimum metabolic rate and cell growth. The progression towards continuous cell production at high volumes, lends itself to new PAT being introduced, that can provide real-time information from a cell culture (PAT, 2022).

Spectroscopy-based approaches have been explored at PAT due to their ability to non-destructively interrogate materials (Esmonde-White et al., 2022). Infrared (IR) spectroscopy is an analytical technique capable of deducing subtle biological changes in samples without the need for extensive sample preparation or expensive reagent. It has been routinely used in the analysis of cells and cell derivatives, as well as in the field of PAT (Helgers et al., 2021). Attenuated total reflection Fourier transform Infrared (ATR-FTIR) spectroscopy in particular has shown advantages in this field due to the robustness of measurements, and ability to analyse aqueous samples (Landgrebe et al., 2010). However, the technique has been mostly restricted to at-line monitoring due to limitations with existing instrumentation, specifically the fixed crystal through which ATR measurements are made. Advancements in traditional instrumentation may allow high-throughput, automated analysis that may be more readily adapted to an in-line system. The use of detachable internal reflection elements that are also disposable, immediately lends itself to rapid at-line analysis, but could be integrated into bioreactor designs or probes for real-time analysis (Butler et al., 2019). An extensive review conducted by Tiernan et al., 2020 outlines the current advances ATR-FTIR spectroscopy within the bioprocessing environment, including looking at metabolite quantification within a bioreactor producing monoclonal antibodies (Wu et al., 2015).

There have been a multitude of studies which have attempted to integrate FTIR as an in-line or on-line process monitoring tool, including the use of FTIR probes which can be submerged directly into a bioreactor to provide real-time measurements (Grimm Marko Sandor et al., 2022; Goldfeld et al., 2014; Clavaud et al., 2013; Alimagham et al., 2021). FTIR probes have been utilised in different sampling modes including near IR, focused beam reflectance as well as mid-IR and ATR (Gerzon et al., 2022). However, it has been shown that FTIR probes may be sensitive to environmental changes such as temperature and humidity, as well as issues with various particle sizes, where fluctuations in these variables can lead to measurement inaccuracies (Dhruv, 2022). They can also suffer from poor spectral peak resolution in the case of the NIR probes where the sample matrix is complex, as well as requiring substantial cleaning and sterilisation prior to use (Arnold et al., 2002; Crowley et al., 2005). The main issue with these probes are materials they are constructed of. They typically have poor physical and transmission characteristics and as such are restricted in length which can be an issue in the manufacturing environment. Additionally these materials vibrate at regions of interest and as such have the potential to obscure important biological peaks (Roychoudhury et al., 2006). There still exists the need for extensive development in this area to make the technology more viable.

A study conducted by Rhiel *et al* monitored the glucose and lactate profiles in CHO cell cultures *in situ*, real-time using an ATR diamond probe. They were able to accurately monitor both metabolites for up to 15 days while keeping the probe *in situ* for 2.3 years without requiring calibration. This work proved the viability of such a technology while utilising *in situ* probes and has supplied a good stepping stone to advance this type of analysis (Rhiel et al., 2002). In contrast, here we explore the use of disposable crystals which have the potential to be integrated into existing vessels to provide an in-line analysis technology, which does not require cleaning or sterilisation, and can measure substances that may not be achievable through using a probe. This would lead to reduced down-time between batches as no cleaning would be required and the risk of cross-contamination would be greatly reduced.

This study describes the use of an innovative ATR-FTIR spectroscopy platform for feasibility testing in the field of bioprocessing. This novel approach overcomes sample matrix issues such as particle size or sample turbidity, removes the need for sterilisation between batches, and has the potential to become a minimally invasive in-line technology. Initially, a qualitative approach is explored to determine cell health by analysing both cells and cell media. Furthermore, a quantitative study to characterise cellular metabolites (glucose and lactic acid) in cell media and to subsequently develop quantification models to predict glucose and lactic acid levels. This data will provide proof-of-concept evidence that a novel ATR-FTIR spectroscopy platform is adept at bioprocess monitoring, in advance of future instrumentation developments using this technology. The results of the qualitative analysis showed the suitability of cell media for obtaining health related information from a bioreactor. Subsequently, this study investigated the monitoring of key growth indicators, glucose and lactic acid, in cell media. These critical process parameters have been quantified in previous studies which have been used to benchmark this study (Rajan Parachalil et al., 2019).

## 2 Materials and methods

### 2.1 Qualitative analysis of CHO cells

In this initial feasibility study, we investigated spectral differences derived from Chinese Hamster Ovary (CHO) cells subjected to different growth environments. CHO cells are a cell line that can be genetically modified to express a range of proteins, including therapeutic drugs and monoclonal antibodies, that are used in the pharmaceutical industry (Omasa et al., 2010). The aim of this study was to investigate if cell and cell media samples could be analysed as indicators of nutrient stress during cell expansion.

#### 2.1.1 Sample preparation

CHO cells obtained from a commercial partner were either cultured in optimum growth conditions (“Healthy”) or allowed to grow passed the optimum time for cell passage (“Nutrient Deficient”). Samples were obtained directly from the cell culture flasks, containing both cells and cell media. Triplicate CHO cell samples were obtained for both “Healthy” and “Nutrient Deficient” samples, and frozen at −80°C in 1 mL cryovials. Prior to analysis, samples were thawed at 37°C, gently inverted, and centrifuged at 1000 rpm for 1 min to concentrate cellular material and to separate cell media. Both the supernatant and the cell pellet were analysed in this study.

Samples were deposited on Dxcover^®^ Sample Slides (Dxcover Ltd., United Kingdom). For more information regarding the Dxcover^®^ Infrared Platform please see Appendix A (Butler et al., 2019). The Sample Slides contain a background well and three sample wells to enable triplicate measurements. Three independent Sample Slides were also prepared per sample. Following sample thawing, 3 μL of sample was immediately deposited onto the surface of a Sample Slide well and allowed to dry for 10 min.

#### 2.1.2 Spectral analysis

The analysis of Sample Slides was facilitated by the Dxcover^®^ Autosampler, which is a novel FTIR spectrometer accessory that automates the movement of the Sample Slide so that each of the background and sample wells can be analysed in turn. The Autosampler was installed in a Perkin Elmer Spectrum Two spectrometer (Perkin Elmer, United Kingdom). Spectra were obtained at a resolution of 4 cm^−1^, with 16 co-added scans and a data spacing option of 1 cm^−1^. In this instance, spectra were cut to 3700–1000 cm^−1^ and vector normalised. Principal component analysis (PCA) was employed as an unsupervised multivariate analysis approach to explore variance in the dataset that may be indicative of differences in the samples analysed. Data analysis was conducted using in-house developed scripts in both Matlab and Python computing languages.

### 2.2 Metabolite quantification in cell media

#### 2.2.1 Sample preparation

Glucose free Dulbecco’s Modified Eagle’s Medium (Sigma, United Kingdom), was prepared with Bovine Serum Albumin (BSA) (Sigma, United Kingdom) to make a 1% BSA in cell media stock solution. BSA was added to aid sample deposition and spreading onto the Sample Slide. Three separate concentration profiles were investigated; 1) glucose (Sigma, United Kingdom), 2) lactic acid (Sigma, United Kingdom) and 3) a combination of both. For the binary classifications 1) and 2), cell media was spiked with glucose and lactic acid using a serial dilution at a concentration range of 100, 80, 60, 40, 30, 20, 10, 8 mg/mL 648 spectra were collected in total for each of these classifications, with 81 spectra per concentration.

For the combined approach, each a negative correlation gradient was created to mimic the environment in a bioreactor (Table 1). A total of 648 spectra were collected in total, with 81 spectra for each concentration.

**TABLE 1:** Combined Glucose and Lactic acid concentration profile.

Glucose concentration (mg/mL)	Lactic acid concentration (mg/mL)
150	50
100	100
80	120
60	140
40	160
30	170
20	180
10	190

Sample Slides were prepared by depositing the sample directly into the centre of the three sample wells. Three replicate Sample Slides were prepared for each concentration of the study. Three independent iterations of the study were conducted, with all results collated during analysis. This was conducted in triplicate across three separate sample runs to capture the variability in sample preparation. Samples of unknown concentrations were also created by a separate analyst to be used as an independent test set.

#### 2.2.2 Spectral analysis

The spectral acquisition parameters of Sample Slides were as previously stated for the previous study. The spectra were pre-processed using an in-house Python-based script. In this instance, spectra were cut to the range of 3700–950 cm^−1^ to ensure all biological information arising from the molecular vibrations of the proteins, lipids and carbohydrates, whilst excluding more variable regions of the spectrum. Spectra were then vector normalised to account for pathlength differences within the samples which can manifest as absorption differences not related to concentration. Partial Least Squares (PLS) was then utilised to create quantitative models to describe the metabolite concentration gradients. PLS is a linear regression model that reduces the dataset dimensionality to allow comparison between variables. The optimum number of components for the models were chosen based on where the *R* ^2^ value stabilised; for the glucose and combined model 5 components were used, and 4 components for the lactic acid model. The PLS model was first trained on a dataset containing 648 spectra in total, with 81 spectra for each concentration. The training data set consists of; 8 concentrations x 3 sample slides (each of which have 3 sample wells which are analysed in triplicate) resulting in 27 spectra per concentration. This process was repeated a further two times to generate three independent data sets to account for experimental variability, resulting in a total of 81 spectra per concentration.

This generated a plot of predicted against observed concentration where the model has seen all training data. A leave-one-concentration-out (LOCO) cross validation was then performed where a single concentration from the training data is used as an internal validation set to assess the model, here the model is only seeing 8 out of 9 of the concentrations at any one time, with the 9^th^ being used as the internal validation set. This process is repeated, leaving out each concentration at least once, and then the full process reiterated 21 times This generates a LOCOCV model with subsequent training and cross-validation statistics (such as *R* ^2^ and RMSECV values).

For the combination approach, a multi-output regression was employed also utilising a subsequent LOCOCV. This was conducted by using a separate dataset that contains the concentrations for both glucose and lactic acid. The multi-output method utilises the same method as the binary models in that it is also a PLS regression, however it uses the both concentrations as an input for the training set. This is termed a multi-output model as the predicted metabolite concentrations are generated simultaneously. The reasoning behind this methodology is so that in a production environment, both profiles can be monitored simultaneously using a singular model.

## 3 Results and discussion

### 3.1 Qualitative analysis of CHO cells

**FIGURE 1:**
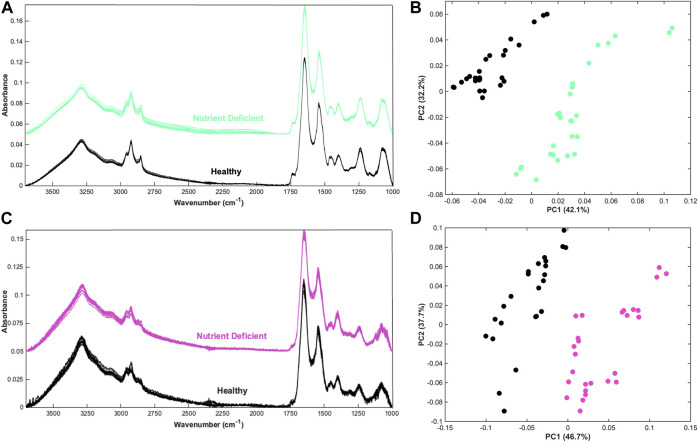
Qualitative analysis of CHO cells and CHO cell media **(A)** Offset spectra from nutrient deficient and healthy CHO cells in pellet form, **(B)** principal component analysis (PCA) scatterplot of the nutrient deficient and healthy cell spectra, **(C)** Offset spectra from corresponding nutrient deficient and healthy the supernatant CHO cell media, and **(D)** PCA of the corresponding nutrient deficient and healthy CHO cell media data.

Spectra obtained using the ATR-FTIR spectroscopy system produced high quality spectra from CHO cells which have been spun down into a pellet sample (Figure 1A). It is noticeable that more variation is evident in the spectra of the “Nutrient Deficient” sample group, particularly in the high wavenumber region and silent region. As these samples were in a nutrient stressed state, it is possible that there was an increase in cell death prior to analysis which may contribute to spectral variability. Principal component analysis (PCA) is able to effectively separate the two sample groups, shown predominantly in principal component (PC) 1 (Figure 1B). The data suggests that changes during cell expansion could be characterised by ATR-FTIR spectroscopy.

Within the production environment the analysis of cell media rather than cellular material could provide an indirect measurement of cell health that does not require removal of valuable samples from a bioreactor. This lends itself more closely with in-line measurements, which could measure the cell environment. Spectra obtained from the corresponding supernatant CHO cell media show clear biological profiles (Figure 1C). Spectra show increased noise throughout the spectra, which can be attributed to a reduced signal on account of the relatively unconcentrated sample type. This could likely be improved with greater sample volume and altering spectral acquisition parameters such as increasing the number of co-added scans to improve the signal-to-noise. As with the cell samples, PCA is able to differentiate the two sample groups along the PC1 axis (Figure 1D). As PCA is an unsupervised approach, the results are promising as spectral differences are discernible without providing class information.

For implementation into bioprocessing pipelines, it would first be necessary to create appropriate reference data, such as described here. This data would be used to establish quality references that could subsequently be used to predict data as it is generated.

## 4 Quantitative analysis of cellular metabolites

The previous results show a suitability of the ATR-FTIR platform for the analysis of cell media. In this study, the ability of the platform to quantify specific molecules in the nutrient media was explored. Infrared spectroscopy can be used to determine the concentration of molecules by analysis of absorbance intensity at spectral peaks inherent to the molecule of interest, following the principle of Beer-Lambert law (Ganzoury et al., 2015). Quantification of molecules can be approached using simple linear modelling of specific wavenumbers, or using multivariate approaches that investigates multiple spectral regions across the spectrum.

### 4.1 Glucose quantification

Glucose is an IR active molecule that has strong absorbance bands across the full spectrum, including a wide O-H band between 3570 and 3120 cm^−1^, the C-H band between 3085 and 3020 cm^−1^, a C-O band between 1230 and 1000 cm^−1^, the C-O-C stretching and deformation between 1275 and 800 cm^−1^ and an intense peak at 1074 and 1033 cm^−1^ arising from C-O (Petibois et al., 1999; Kosa et al., 2017). 

**FIGURE 2:**
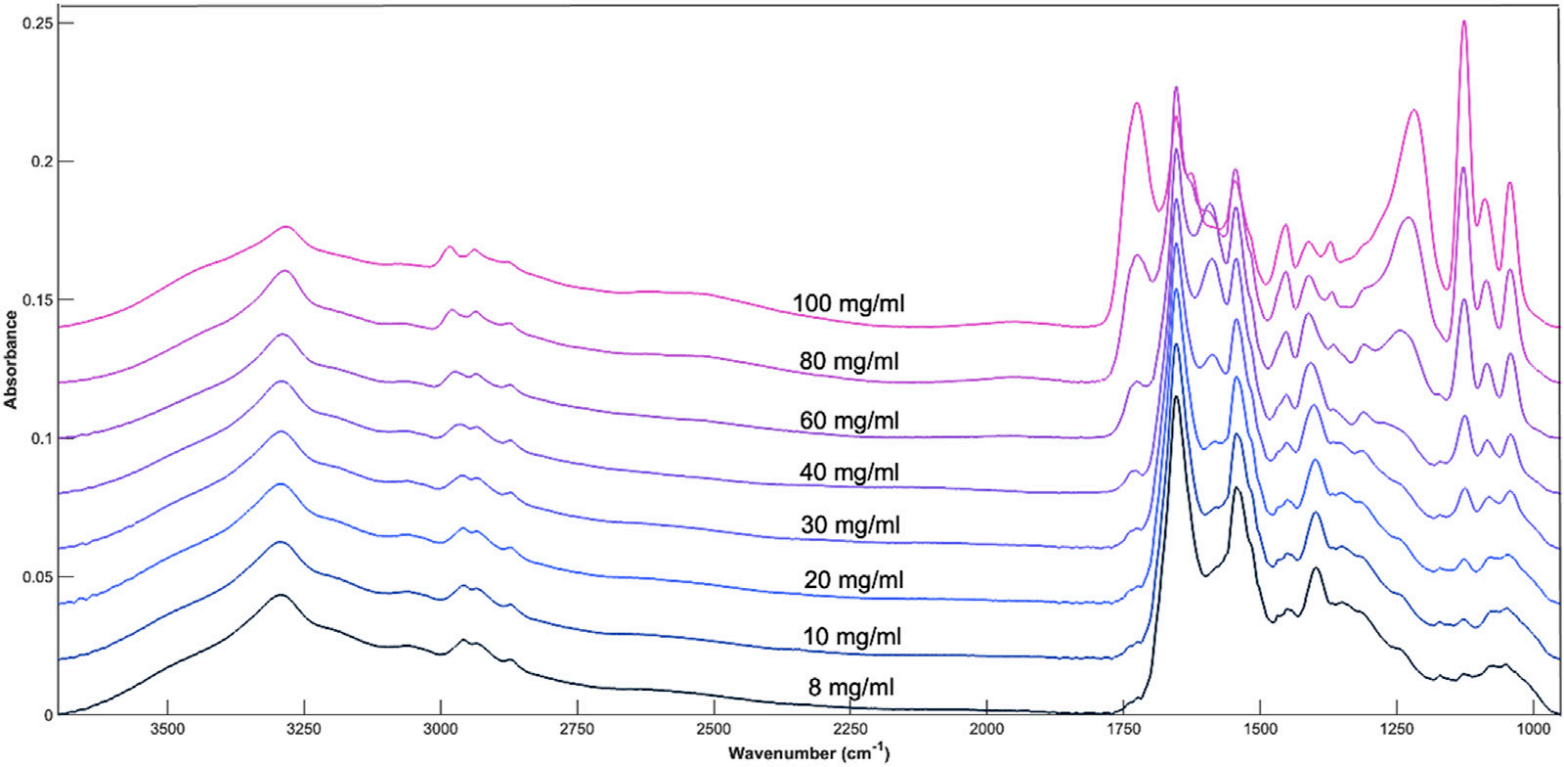
Average spectral plot of DMEM cell media with increasing concentrations of glucose. 8, 10, 20, 30, 40, 60, 80, and 100 mg/mL.

The influence of glucose when spiked into cell media is most noticeable at both the 1074 and 1033 cm^−1^ peak, corresponding to the C-O stretch of glucose (Figure 2). A moderate change in intensity can also be seen at other glucose related peaks between 1200 cm^−1^ and 900 cm^−1^ which shows the concentration of glucose within the cell media solution can be monitored by the intensity of the absorbance bands. As glucose related peaks contribute to the full spectrum, this may also affect the absorbance at the Amide I and II peaks (1654 cm^−1^ and 1545 cm^−1^) which are largely representative of cell media and BSA.

**FIGURE 3:**
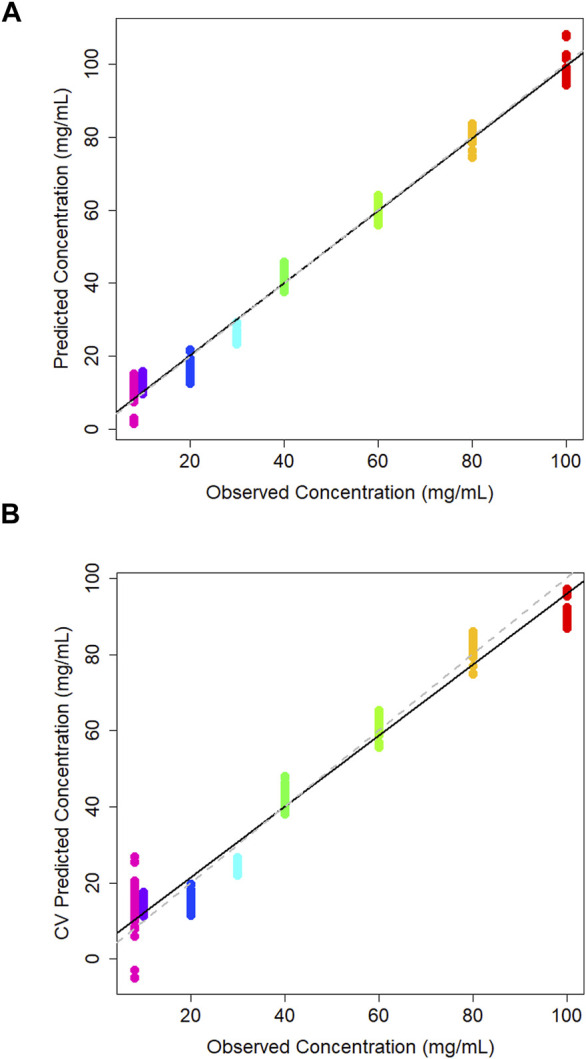
**(A)** PLS model of predicted vs. observed concentration of glucose in DMEM cell media generated using a partial least squares model, and **(B)** corresponding leave-one-out cross validation partial least squares model.

A PLS regression model was used to create a concentration calibration plot (Figure 3A).

The leave-one-out cross-validation results of the training data give realistic view of the model accuracy, which treats one of the classes as an unknown and attempts to predict it based on the training data supplied (Figure 3B). Typically, the *R* ^2^ for this model is less than the PLS model. The *R* ^2^ value for the cross-validation model is 0.969 which is promisingly close to the PLS model statistics which has seen all of the training data, indicating model stability. The Q^2^ value was 0.956 and the root mean square error estimated from cross-validation (RMSECV) is 5.7 mg/mL. Similarly to the PLS model the sample spread is increased at lower concentrations as the limit of detection (LOD) is approached, making it harder to predict concentrations in this range. This concentration range has been selected in order to encompass a variety of product being created within the industry which can range from concentrations starting at 1 mg/mL up to 100 mg/mL for some applications (Kosa et al., 2017). Here, the concentrations above the 10 mg/mL the groupings are tightly clustered which suggests consistency across the three independent sample sets. LOOCV models also tend to predict worse at the end-points as at some point that concentration must be left out, which makes it hard to extrapolate the data past these points which may be attributing to the variation at the lower extremities.

### 4.2 Lactic acid quantification

**FIGURE 4:**
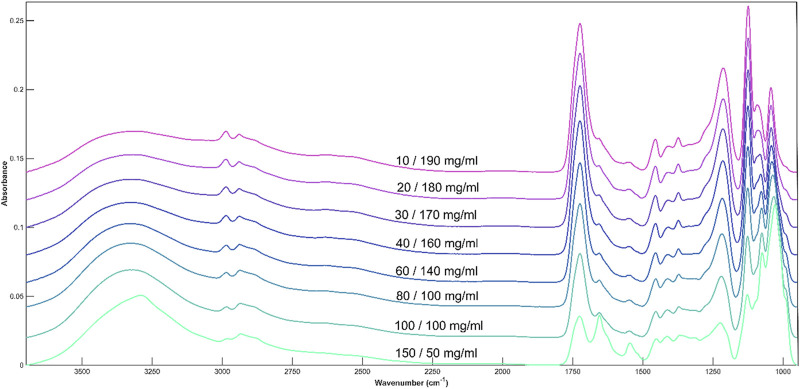
Average spectral plot of DMEM cell media with increasing concentrations of lactic acid. 8, 10, 20, 30, 40, 60, 80, and 100 mg/mL.

Lactic acid also exhibits infrared peaks across the full 3700–950 cm^−1^ wavenumber range, particularly in the fingerprint region where bands corresponding to the C-O and C-O-C bonds of carboxylic acids are present, as well as C-H bending peaks. The peak at 1215 cm^−1^ can be assigned to the C-O stretching peak and the 1730 cm^−1^ peak corresponds to the C=O stretching of the carboxylic acid (Păucean et al., 2017). These are two peaks which vary the most in terms of intensity within the spectrum and show the correlation between peak intensity and concentration of analyte (Figure 4).

The mean spectral plot for lactic acid shows more variance overall compared to glucose, particularly across the fingerprint region. At the peaks between 1200 and 1000 cm^−1^ the spectra of lower concentrations, such as 8, 10, and 20 mg/mL converge in peak intensities which indicates that the limit of detection may be approached at around these values. Again, these concentrations have been selected based on previous studies within this area looking at a range between 1 and 100 mg/mL (Tamburini et al., 2014).

**FIGURE 5:**
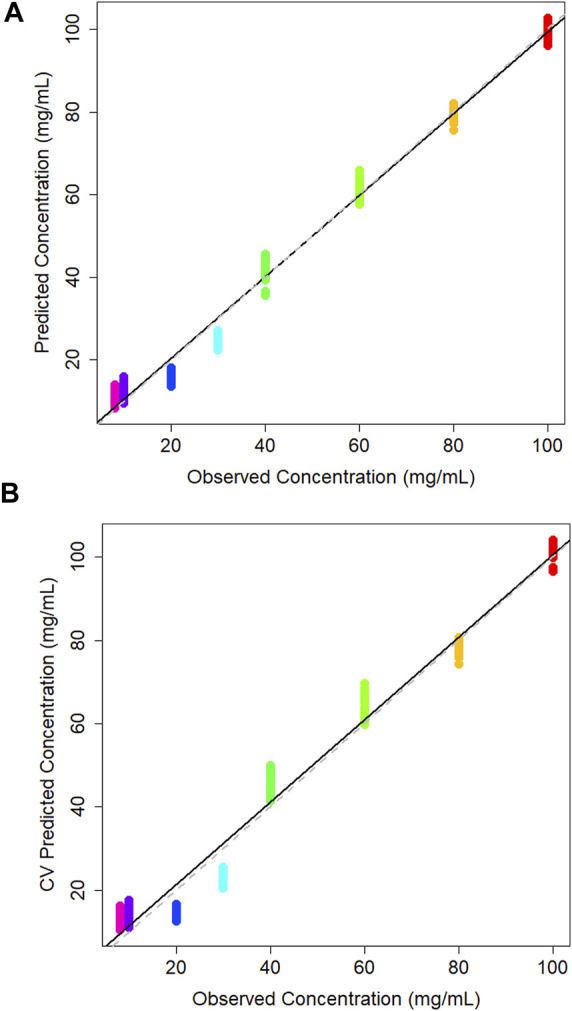
**(A)** Calibration plot of predicted vs. observed concentration of lactic acid in DMEM cell media generated using a partial least squares model, and corresponding **(B)** cross validation partial least squares calibration plot.

A PLS model was used to generate a plot of predicted against observed concentration where the model has seen all of the training data. This yielded an *R* ^2^ value of 0.989, again indicating the regression line is approximating the data well (Figure 5A). The 20 and 30 mg/mL samples are predicted slightly lower than their true concentration, but the rest of the samples across the full range are predicted close to their observed concentrations. All sample groups are tightly clustered together with minimal intra-sample variability.

The leave-one-out cross validation model yields an *R* ^2^ value of 0.976, a Q^2^ value of 0.944 and an RMSECV of 5.04. As with the PLS model, samples 20 and 30 mg/mL are predicting lower than their true concentration (Figure 5B). It is reassuring that there is minor intra-sample spread across all concentrations. This may indicate that the ATR-FTIR approach is consistently able to measure lactic acid in the sample medium.

### 4.3 Glucose and lactic acid

**FIGURE 6:**
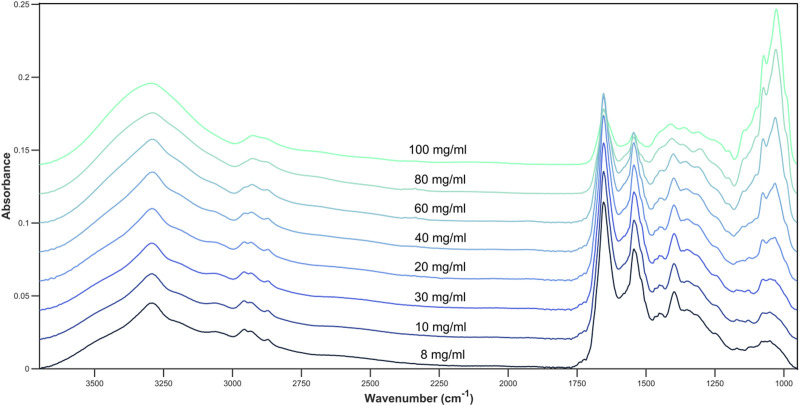
Average spectral plot of DMEM cell media with alternating concentrations of glucose and lactic acid.

The combination of both metabolites produces a spectrum which contains characteristic peaks corresponding to each compound. The peaks are well defined, and the overall spectrum has a high signal-to-noise ratio which allows for easy identification of individual bands (Figure 6). The spectra are dominated by the 1730 and 1215 peaks when lactic acid is found in high concentrations The Amide I and II peaks are only visible at 150 mg/mL glucose and 50 mg/mL lactic acid. Even larger in intensity than the lactic acid peaks are the two glucose peaks at 1125 and 1030cm^−1^.

**FIGURE 7:**
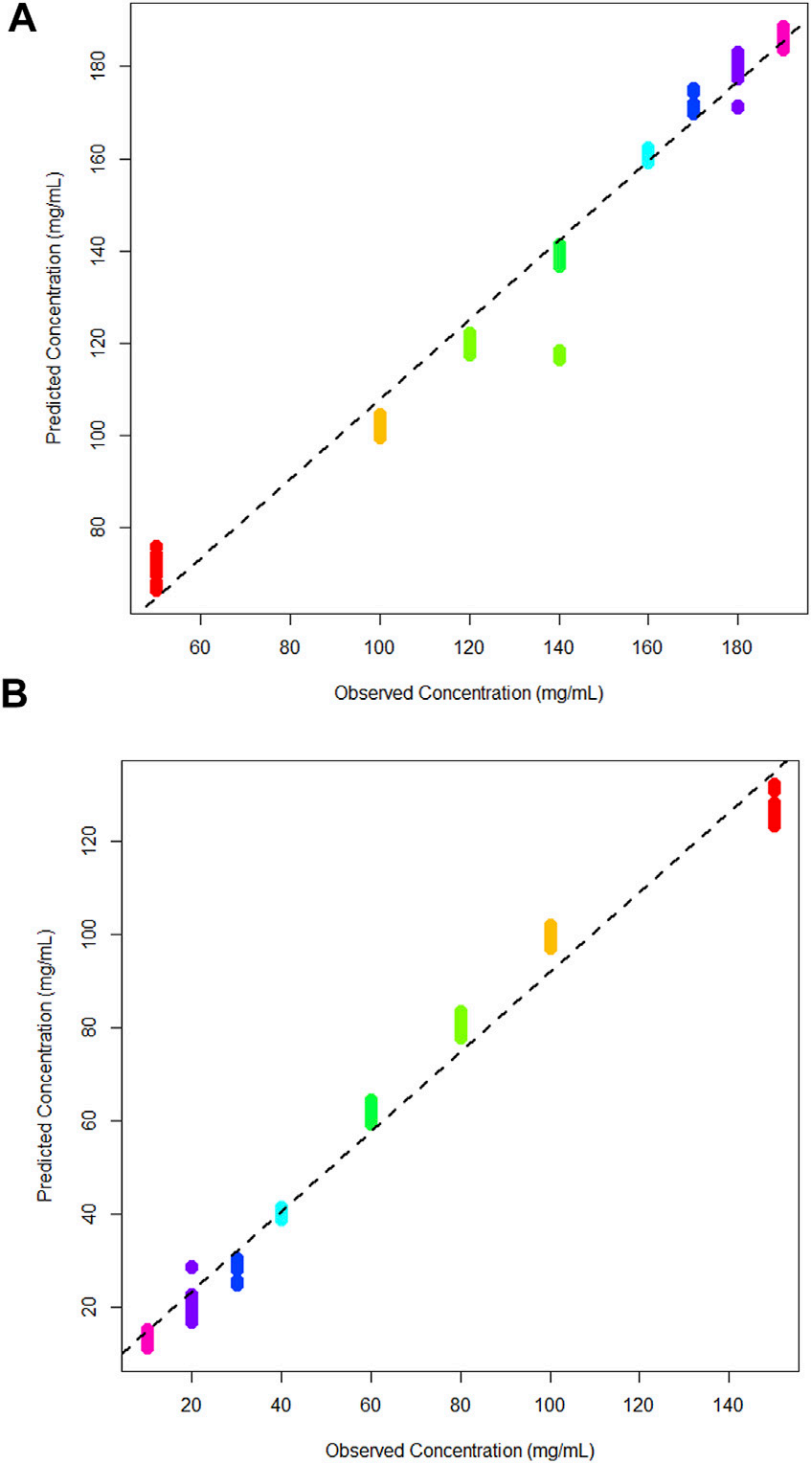
**(A)** Cross validation multi-output partial least squares models for glucose, and **(B)** lactic acid in DMEM cell media.

A multi-output PLS model was trained to quantify the concentration of glucose and lactic acid simultaneously. As previously, a leave-one-out cross validation was performed to assess the model for both analytes (Figures 7A, B). The *R* ^2^ value for both was 0.98, meaning the multi-output PLS model was estimating the concentrations with a high level of precision. Table 2 tabulates the *R* ^2^ and Q^2^ results for each model. Overall, each concentration is tightly clustered showing reproducibility over three independent tests.

**TABLE 2:** *R* ^2^ and Q^2^ values for the cross-validation models of all models.

Model	*R* ^2^	Q^2^
Glucose	0.969	0.956
Lactic Acid	0.976	0.944
Glucose and Lactic Acid	0.980	-

Figure 5B shows the cross-validation plot for lactic acid. There is a significant set of samples for the 140 mg/mL group that are lower concentrations than expected. However, the *R* ^2^ result remains high and on par with the glucose quantification. A higher concentration range was evaluated here due to poor predictions of a blind tests set in the binary quantification. 

Thus, the concentration range investigated was expanded to assess whether these predictions would be improved for the combined model.

The multi-output model generated predictions for both analytes in parallel, which would be the most useful application of this technology in the field as it would allow the simultaneous monitoring of analytes within a bioreactor. The model described above was utilised to predict blind test data which has varying concentrations of glucose and lactic acid (Table 3). In this instance both analytes are predicted closely to their expected concentrations. For the glucose predictions, the mean predicted concentrations are generally higher than the expected concentrations, except in the prediction of the 92 mg/mL sample which is accurately predicted. The typical accuracy of current techniques for glucose and lactic acid quantification is between 5% and 10% of the true concentration (Gerzon et al., 2022; Kastenhofer et al., 2021). It is however important to note that specific accuracy requirements may vary depending on factors such as the criticality of the metabolites, the complexity of the bioprocess, and the sensitivity of the analytical method used for measurement.

**TABLE 3:** Expected and predicted concentrations of glucose and lactic acid as determined by partial least squares model.

Sample	Expected concentration (mg/mL)		Mean predicted concentration (mg/mL)	
	Glucose	Lactic acid	Glucose	Lactic acid
1	138	47	151 ± 0.76	48 ± 0.75
2	34	128	46 ± 2.38	153 ± 2.32
3	92	113	92 ± 0.60	108 ± 0.63

The model in this instance may be overestimating extremes in concentration relative to the training dataset. As these extremes are at the top end of the concentrations observed within the bioprocessing environment, a wider error within this range is not of great concern.

With regards to lactic acid measurements, the mean predicted concentrations are close to their expected concentration. The second sample is predicted 12 mg/mL higher than its expected concentration, which is a moderate overestimation by the model. An increased standard deviation for this sample may indicate increased variability during the experimental measurements, for example, the sample slide deposition could have been inconsistent.

Overall, the trends in the table indicate that the combined predictive model shows promise but requires further research to develop accurate predictive models which can easily be transferred into real-world environments. To aid this problem, more concentrations could be analysed and fed into the training model particularly at lower end where the limit of detection is being neared. It is also important to discuss with stakeholders the level of accuracy needed for such an approach. The relatively low standard deviation values for both glucose and lactic acid suggest that the data derived from the platform and the performance of the predictive model is consistent across different samples. However, it is important to note that further development would be required to validate the accuracy and reliability of the model fully.

## 5 Conclusion

The results of this proof-of-concept study suggest that disposable IREs have potential in applications of bioprocessing analytics. For qualitative analysis, the ATR-FTIR based system generated high quality spectra that was able to differentiate cells in a nutrient deficient state compared to control samples. It was also shown that measurements from cell media were able to highlight these differences. This may present a preferable option to in-line monitoring in future, where the valuable materials within the bioreactor can remain undisturbed.

The feasibility of cell metabolite quantification offers a straightforward, rapid, and cost-effective method for monitoring batch health in a bioreactor. The relationship between absorbance and concentration lends itself well to potential process analytical technology, as FTIR is a non-invasive technique. The results demonstrate strong reproducibility across multiple sample runs, indicating the methods robustness against equipment variability. The high *R* ^2^ values observed in the cross-validation models for both binary measurements and combined data underscore the potential for utilizing this approach either at-line or on-line within the manufacturing environment. This technology enhances the current understanding of the cell growth process by offering a ways to accurately track the consumption and expulsion of metabolites within a bioreactor such that the progress and health of a batch can be monitored and adjusted in real-time. In comparison to current methods such as Raman spectroscopy, high-performance liquid chromatography (HPLC) and liquid chromatography mass-spectrometry (LC-MS) which can suffer from analysis issues due to sample matrix, higher complexities and with regard to the chromatographic techniques, longer analysis times. These can be mitigated by utilising this type of ATR-FTIR approach.

It is clear that further development is required to ensure robustness of models for real-world applications. It is likely that training data would need to be generated at site, which is universally accepted to be the standard approach in the field. A benefit of using a disposable sample slide coupled with an ATR-FTIR spectroscopy system is that sample analysis and throughput can be improved by automated analysis which to date has not been possible.

While this study focused on at-line monitoring, future instrumentation developments hold promise for broader applications. The versatile nature of the IREs opens the possibility of incorporating them into bioreactor systems as part of a feedback loop or a sampling chamber with a silicon internal reflection element mounted on the bioreactor wall, enabling real-time measurements as samples pass through. Furthermore, there is exciting potential for at-line measurements, utilizing existing sampling techniques and implementing the Dxcover system as-is within the manufacturing environment to swiftly assess batch health and yield. This opens doors for efficient and practical implementation in the biomedical research and manufacturing fields.

There is scope to apply this novel ATR-FTIR approach across the bioprocessing field as ATR-FTIR spectroscopy as a whole is adaptable and easy to implement. For example, it can be integrated into in-line instrumentation which could be inserted into a vessel or reactor to monitor and control processes as well as determining the endpoint. The technology discussed here is better suited to these applications due to its disposable and flexible nature whereby no sterilisation is required between sample runs, and instrumental considerations such as probe material type, length or distance between reactor and spectrometer are not a factor.

This method can also be adapted to offline measurements where samples are collected and analysed in a laboratory setting which could be useful for quality control measurements to ensure batch to batch consistency. The kinetics of bioprocesses could also be monitored by conducting time-course experiments with frequent sampling intervals. Coupling this technique with interrogative multivariate data analysis opens an avenue to allow for the routine use of ATR-FTIR in the bioprocess environment.

## Data Availability

The datasets presented in this article are not readily available because Dataset belongs to Dxcover Ltd. Requests to access the datasets should be directed to loren.christie@dxcover.com, holly.butler@dxcover.com.

## Appendix A

The Dxcover Infrared Platform—A novel ATR-FTIR spectroscopy platform that allows higher throughput analysis of samples due to disposable and detachable internal reflection elements.
